# Prevalence of Hepatitis E Virus Antibodies, Israel, 2009–2010

**DOI:** 10.3201/eid2104.140245

**Published:** 2015-04

**Authors:** Orna Mor, Ravit Bassal, Michal Michaeli, Marina Wax, Daniela Ram, Oranit Cohen-Ezra, Dani Cohen, Ella Mendelson, Ziv Ben-Ari, Tamy Shohat

**Affiliations:** Central Virology Laboratory, Ministry of Health, Ramat-Gan, Israel (O. Mor, M. Michaeli, M. Wax, D. Ram, E. Mendelson);; Israel Center for Disease Control, Ministry of Health, Ramat-Gan (R. Bassal, T. Shohat);; Sheba Medical Center, Ramat-Gan (O. Cohen-Ezra, Z. Ben-Ari);; Tel-Aviv University, Tel-Aviv, Israel (D. Cohen, E. Mendelson, T. Shohat)

**Keywords:** Anti-HEV, hepatitis E, hepatitis E virus, HEV, viruses, antibodies, IgG, Israel, prevalence, seroprevalence

## Abstract

We investigated prevalence of hepatitis E virus in a sample of the population of Israel. The overall seroprevalence of antibodies to the virus was 10.6% (95% CI 8.4%–13.0%); age-adjusted prevalence was 7.6%. Seropositivity was associated with age, Arab ethnicity, low socioeconomic status, and birth in Africa, Asia, or the former Soviet Union.

Acute viral hepatitis is caused by hepatitis E virus (HEV), which can be divided into 4 genotypes (*1*–*4*). Genotype 1, transmitted mainly through the fecal-oral route, is most common in developing countries in Asia, Africa, and Central America, where the disease is highly endemic. In industrialized countries, hepatitis E is infrequent, although in recent years, sporadic cases have been reported, resulting mainly from zoonotic transmission of HEV genotype 3 (*1**–**3*).

Prevalence rates of HEV IgG, a marker of previous exposure to HEV, range from 1% to 20% in industrialized countries (*1*). This marked variability in reported prevalence has been attributed to the use of different IgG assays with significantly different sensitivities (*4*).

The population of Israel comprises 2 major population groups, Jews (80%) and Arabs (including Muslims, Christians, and Druze); each has distinct cultural and socioeconomic features (*5*). In a study published in 1995, a low seroprevalence of anti-HEV antibodies in Israel was found among Jews (2.81%) and Arabs (1.81%) (*6*). These data were obtained by using an immunoassay developed 20 years ago; newer assays are considered more sensitive and specific to HEV IgG (*4*).

## The Study

We used an age-stratified sampling design to systematically select 729 serum samples from those deposited during 2009–2010 at the National Serum Bank of the Israeli Center of Disease Control. Included were anonymous residual samples from diagnostic laboratories (403 samples) and from healthy blood donors (326 samples). Demographic information (place of birth, population group, and place of residence) was available for all donors. Socioeconomic status (SES) was assessed by the socioeconomic rank as defined by the Israel Central Bureau of Statistics; this system categorizes each place of residence on a 10-point scale based on parameters such as financial resources, housing density, motorization, education, and employment profile (*7*). Levels of HEV IgG were determined by using the DS-Anti-HEV-IgG kit (Diagnostic Systems Italy, Saronno, Italy), which is capable of detecting antibodies against all 4 HEV genotypes. Statistical analyses included χ^2^, *t* test, and univariate and multivariate logistic regression models to evaluate factors associated with hepatitis E infection. Odds ratios (OR) and 95% CI were calculated. A p value of <0.05 was considered statistically significant. Data were analyzed by using SAS version 9.1.3 (SAS Institute, Cary, NC, USA). This study was approved by the Institutional Review Board of Sheba Medical Center (approval no. 9927–12-SMC).

Of 729 samples, 77 (10.6%, 95% CI 8.4%–13.0%) tested positive for HEV IgG. The calculated age-adjusted prevalence rate for the population of Israel was 7.6%. Seropositivity increased significantly with age (Table 1); seroprevalence among persons ≥ 60 years of age was 37.5%, compared with 0.5% among those <20 years of age. HEV seropositivity, which was mainly observed in older persons, was significantly higher among Arabs (22.5%) compared with non-Arabs (10.3%). Among Jews, a significant association was found between samples testing positive for anti-HEV IgG and having been born in Africa (50%), Asia (53.8%), or the former Soviet Union (17.9%) compared with Israel (OR 10.4, 95% CI 6.1–17.9; p<0.0001) and also with an earlier year of immigration to Israel (OR 2.5, 95% CI 1.2–5.4; p = 0.02). The odds for testing HEV IgG–positive were highest among those of low SES (OR 2.9, 95% CI 1.4–5.9; p = 0.003). The multivariate logistic regression model also showed significant association between HEV seropositivity and advanced age, low SES, Arab ethnicity, and having been born in Asia, Africa, or the former Soviet Union (Table 2).

**Table 1:** Prevalence of antibodies to hepatitis E virus categorized by study population demographics, Israel, 2009–2010*

**Characteristics**	**No. samples tested**	**HEV-seropositive samples**		**Odds ratio (95% CI)**	**p value**
		No.	% (95% CI)		
Age, y					
<20	212	1	0.5 (0.01–2.6)	Reference	
20–39	189	2	1.1 (0.1–3.8)	2.3 (0.2–25.1)	0.51
40–59	168	14	8.3 (4.6–14.0)	19.2 (2.5–147.4)	0.005
≥60	160	60	37.5 (30.0–45.5)	126.6 (17.3–926.6)	<0.0001
Sex					
M	394	38	9.6 (6.9–13.0)	Reference	0.38
F	335	39	11.6 (8.4–15.6)	1.2 (0.8–2.0)	
Birthplace					
Israel	518	26	5 (3.3–7.3)	Reference	<0.0001
Africa, FSU, Asia	121	43	35.5 (27.-44.8)	10.4 (6.1–17.9)	
Population group					
Jews	562	58	10.3 (7.9–13.1)	Reference	0.002
Arabs	80	18	22.5 (13.9–33.2)	2.5 (1.4–4.6)	
Year of immigration					
<1970	89	37	41.6 (31.2–52.5)	2.5 (1.2–5.4)	0.02
1970–1989	31	2	6.4 (0.8–21.4)	0.2 (0.1–1.2)	0.08
1990–2000	54	12	22.2 (12.0–35.6)	Reference	
>2000	13	1	1 (0.2–36.0)	0.3 (0.03–2.5)	0.26
Socioeconomic status					
High, ranks 7–10	203	12	5.9 (3.1–10.1)	Reference	
Intermediate, ranks 4–6	211	25	11.8 (7.8–17.0)	2.1 (1.0–4.4)	0.03
Low, ranks 1–3	193	30	15.5 (10.7–21.4)	2.9 (1.4–5.9)	0.003
Total	729	77	10.6 (8.4–13.0)	NA	NA

*FSU, former Soviet Union; NA, not applicable.

**Table 2:** Multivariate logistic regression analysis for factors associated with anti HEV seropositivity, Israel, 2009–2010*

Characteristics

Odds ratio (95% CI)

p value

Age, y

<20

Reference

20–39

1.5 (0.1–24.3)

0.79

40–59

17.6 (2.2–143.8)

0.008

≥ 60

100.1 (12.1–830.3)

<0.0001

Socioeconomic status

High, ranks 7–10

Reference

Intermediate, ranks 4–6

2.1 (0.7–5.7)

0.16

Low, ranks 1–3

3.4 (1.1–10.1)

0.03

Population group

Jew

Reference

Arab

7.1 (2.1–24.0)

0.002

Country of birth

Israel

Reference

Africa, FSU, Asia

3.8 (1.4–10.8)

0.1

*FSU, former Soviet Union.

The prevalence of HEV antibodies we found is higher than previously reported in Israel and is consistent with other studies that have reported higher prevalence rates with the use of new, more sensitive immunoassays (*4*). Although it was argued that high prevalence of anti-HEV antibodies could be attributed to nonspecific or false-positive serum reactions (*8*), the low prevalence we found among those <40 years of age and the significant association between the prevalence of HEV antibodies and older age suggest that it is unlikely to be a result of nonspecific serum reactions.

Association of seropositivity with age was also reported in Denmark and the United Kingdom (*9**,**10*). Such associations could represent a cohort effect related to infection in the past or could be a result of ongoing low incidence of HEV infection resulting in cumulative exposure to infection over time. In Denmark, a statistically significant difference was detected in the overall HEV prevalence among samples from blood donors collected in 1983 versus those collected in 2003, suggesting that past exposure contributed to the anti-HEV response and that the prevalence of HEV seropositivity had decreased over the years (*9*). In contrast, in HEV-endemic countries, transmission might be ongoing; in India, the age-specific prevalence of anti-HEV did not change during 1982–1992 (*11*). Similarly, overall, 3.2% of blood donors in France were HEV-positive, but 52.5% of blood donors in southwest France, which includes the Midi-Pyrénées region, where HEV is endemic, were HEV positive (*12*). 

No HEV outbreak has been documented in Israel; the total number of autochthonous HEV infections is unknown. Acute infections were reported only among travelers returning to Israel from HEV-endemic countries (*13*), further suggesting that ongoing transmission of HEV in Israel is unlikely.

Our findings that low SES is associated with HEV seropositivity is supported by others who have suggested low SES and poor environmental conditions are risk factors for HEV infection (*14*). The higher seropositivity observed among persons born in Africa, Asia, or the former Soviet Union corroborates with HEV endemicity and documented large outbreaks in these regions (*3*).

Although we found the seropositivity rate in the population of Israel to be higher than previously reported, and associated with specific population subgroups, this study has several limitations. Being a cross-sectional study, it is impossible to rule out recent or ongoing infections among older persons. To better address this issue, the presence of HEV RNA and anti HEV IgM antibodies, which together provide the most sensitive measure for acute infection, should be assessed in a much larger representative sample of older persons of all populations. Moreover, HEV genotype which was not addressed in this study should be determined in persons positive for HEV RNA. Because the prevalence of HEV infection in animals in Israel has not been documented, a study to better understand the HEV transmission root would be useful, especially because the consumption of pork, a common source of HEV infection, is religiously prohibited for both Arab Muslims and Jews (*15*).

## Conclusions

The findings of this study indicate high numbers of past HEV infections among immigrants to Israel, which seemingly occurred in countries in which HEV is endemic. The higher HEV prevalence found among older persons in the Arab population, which was also associated with low SES, compared with the non-Arab population could be attributed to previous local exposure. The current overall HEV infection rate in Israel is low, as suggested both by the low prevalence in the younger population and the absence of any documented HEV outbreaks in Israel. However, the nature of this study cannot rule out ongoing infections among older persons. Surveillance of HEV through mandatory reporting of communicable diseases should continue to enable detection of emerging and reemerging infections.
